# Commentator Discussion: Impact of left ventricular rehabilitation on surgical outcomes in patients with borderline left heart hypoplasia

**DOI:** 10.1016/j.xjon.2024.10.020

**Published:** 2024-10-29

See Article page 359.

Presenter: Haonan Cheng

**Dr Sitaram Emani***(Boston, Mass)*. Thank you very much. That was a very nice presentation and very interesting data. I think the one obvious data point that's missing is the clinical behavior of the patients who were in this cohort who underwent biventricular repair. And I guess my question to you is, does this matter? Does mitral valve growth correlate with clinical outcomes? If you did not have mitral valve growth, did they present with left atrial hypertension, mitral valve stenosis, pulmonary hypertension, mortality?

**Figure:**
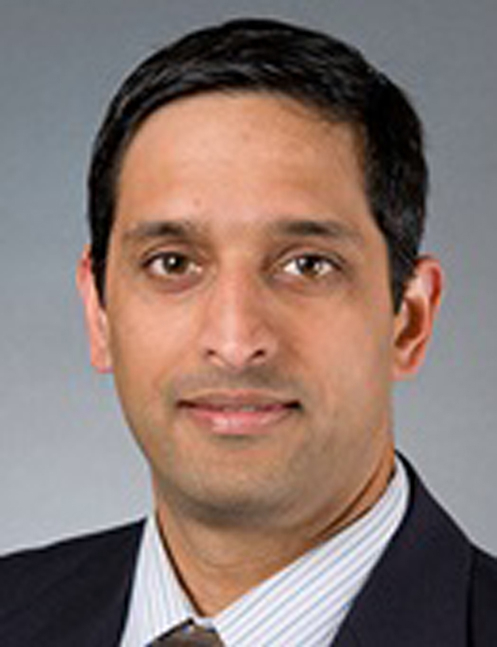

**Ms Haonan Cheng***(Munich, Germany)*. I think our study cannot answer this question because our study or our patients did not present with left atrial hypertension after 6-month follow-up. We want to indicate that unrestrictive atrial septal defect (ASD) could be—in patients with restrictive ASD, there could be a less potential for future mitral valve growth. So, in patients who had small mitral valves, with high ASD pressure gradient, it might not be prudent to proceed to biventricular (BiV) repair in neonatal status. We may need to prolong our observation time and see whether there is a growth potential for the left ventricle. But in patients who had unrestrictive ASD with a small mitral valve, but maybe not too small—[*inaudible*] is less than minus 4 or minus—or less than minus 3, then there is a greater chance for a successful BiV repair. So, we cannot reach the conclusion that we can have a better clinical outcome. But we want to indicate that these conditions might be a parameter for us to decide whether the patient is a good candidate for BiV repair or not in the neonatal status.

**Figure:**
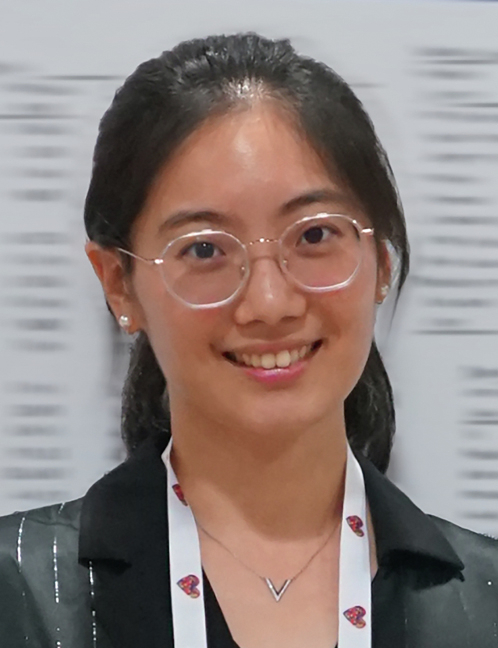

**Dr Emani**. Thank you. It sounds like none of these patients died, at least, at 6 months.

**Ms Cheng**. Yes.

**Dr Emani**. And so, I think the hypothesis that this is clinically relevant may be something that you have to look at because it comes back to the question—I mean, if we're saying that if you have a gradient across ASD, that you should not do a biventricular repair. That's a strong statement, and most of us would say, “Yeah. If you have a little bit of a gradient across your ASD, if your ASD is restrictive, that valve will grow over time.” This leads me to my next point, for which I'm sure you don't have data. But what happens to the growth of the mitral valve beyond 6 months? Because 6 months is kind of an acute loading change. What we really care about is when does that valve become so fixed that it won't grow in the future. And do we know that 6 months is a relevant time point?

**Ms Cheng**. I think we'll look up a little bit longer in our follow-up, but so far, we don't have that data right now.

**Dr Emani**. Yeah, I think it's just in terms of comparing with the other group on which we didn't spend a lot of time, the stage I Norwood patients. Clearly, the mitral valve did not grow over time, and so that's an important thing to consider because if you want to recruit those patients you do have to come back and restrict the atrial septum. It's clear that if you don't have atrial septal promotion of flow, you won't grow that mitral valve. Very nice data. Thank you.

**Ms Cheng**. Thank you.

**Unidentified Speaker 1**. One more question.

**Unidentified Speaker 2**. Sorry. Great talk. So, did you guys look at the morphology of the septum and the changes in pressurization? Meaning, is there any possibility that at least part of the growth that you're seeing is actually just the morphology of just pressurizing a left ventricle in a biventricular repair? And actually, having more expansion of that mitral valve that you don't see necessarily in the single ventricle where both ventricles are actually just pressurized, and you have that compression of the mitral valve?

**Ms Cheng**. Well, actually I personally look up all echo cardiac data, but I did not quantify this kind of finding in my analysis. But to my knowledge, also experience, I think these are not results of pressurized, at least, the chamber did look like the left ventricular dimensions grew symmetrically after the repair.

**Unidentified Speaker 1**. Thank you very much.

[*applause*]

## Conflict of Interest Statement

The authors reported no conflicts of interest.

The *Journal* policy requires editors and reviewers to disclose conflicts of interest and to decline handling or reviewing manuscripts for which they may have a conflict of interest. The editors and reviewers of this article have no conflicts of interest.

